# Reduced Inspiratory Muscle Strength in Patients with Type 2 Diabetes Mellitus and Obstructive Sleep Apnoea

**DOI:** 10.1155/2017/4121794

**Published:** 2017-09-25

**Authors:** Thomas Rehling, Anne Margareta Banghøj, Marie Hvelplund Kristiansen, Lise Tarnow, Stig Molsted

**Affiliations:** ^1^University College (UCC) Department of Physiotherapy, Hillerød, Denmark; ^2^Department of Clinical Research, Nordsjællands Hospital, Hillerød, Denmark; ^3^Department of Cardiology, Nephrology & Endocrinology, Nordsjællands Hospital, Hillerød, Denmark; ^4^Health, Aarhus University, Aarhus, Denmark

## Abstract

**Background:**

Obstructive sleep apnoea (OSA) is related to type 2 diabetes (T2DM), and it may be associated with reduced inspiratory muscle strength (IMS). The aim of this study was to investigate the IMS in patients with T2DM, with or without OSA.

**Methods:**

Patients with T2DM with OSA (*n* = 33) and without OSA (*n* = 28) were included. The maximum IMS was tested using the POWERbreathe KH2 device. Reference IMS values were data calculated using an algorithm based on general populations and adjusted for age and gender.

**Results:**

There was no difference in IMS between the OSA group (median (range) 77 (35–124) cmH_2_O) and the non-OSA group (84 (33–122) cmH_2_O) (*p* = 0.97). The IMS values were reduced in the OSA group compared with the reference values (92.9 (62.3–100.0) cmH_2_O) (*p* = 0.030), whereas the non-OSA group did not have reduced IMS. When the IMS values of all T2DM patients were compared with reference values, the IMS values were 79 (33–124) cmH_2_O and 93.8 (62.3–102.4) cmH_2_O, respectively (*p* = 0.017).

**Conclusion:**

No difference in IMS between patients with T2DM with or without OSA was found. However, patients with T2DM and OSA had reduced IMS compared with age- and gender-matched references whereas the non-OSA group did not have reduced IMS.

## 1. Introduction

Physical activity (PA) is a corner stone in the treatment of type 2 diabetes (T2DM). The effects of PA on patients with T2DM include an improved glycaemic control, reduced blood pressure, an improved blood lipid profile, and a reduced waist circumference [1–4]. Thus, PA may have the potential to decrease the risks of diabetic complications and mortality in patients with T2DM. As PA is an inexpensive and nonpharmacological treatment, the nature of PA further potentiates its therapeutic appeal. However, the level of PA in patients with T2DM remains reduced as compared to that in subjects without T2DM [5].

Patients with T2DM suffer from several complications and comorbidities; among them is obstructive sleep apnoea (OSA) [6]. The prevalence of OSA among patients with T2DM has been reported to be 23–87% [6] which is in contrast to previous data from the general population reporting an OSA prevalence of 1–4% [7, 8]. Obstructive sleep apnoea is associated with an increased risk of cardiovascular disease [9]. In addition, OSA is associated with excessive daytime sleepiness, mood changes, and cognitive dysfunction [10], all conditions that may impair the patients' ability to increase their PA levels and make other important lifestyle changes. Chasens and Olshansky [11] found that in patients with T2DM, sleepiness was a daily burden and only a minimum number of activities was performed during daytime. These daily activities may also include PA; thus, OSA and excessive daytime sleepiness in people with T2DM may have important negative implications [11].

In a study from 2014, Chien et al. found reduced strength in *m. diaphragma* in patients with OSA without T2DM when compared with matched controls [12]. In another study from 2016, Vranish and Bailey found that six-week inspiratory muscle training (IMT) in patients with OSA without T2DM was associated with positive effects on patient-reported sleep quality and blood pressure [13].

Whilst IMS may be reduced in patients with OSA without T2DM, it is unknown whether this association also is found in patients with OSA with concurrent T2DM. Accordingly, we tested the hypothesis that IMS in adults with T2DM without OSA is greater than that in adults with T2DM and OSA. The aim of this study was to compare IMS in patients with T2DM with and without OSA and to compare the patients' IMS data with the IMS data in a gender- and age-matched reference population.

## 2. Methods

The data from the present study were obtained during the period of August 2015 to January 2017 as part of an ongoing study that aims to investigate the impact of continuous positive airway pressure (CPAP) treatment on arterial stiffness in patients with T2DM and newly diagnosed OSA.

### 2.1. Participants

Patients were recruited from the Department of Cardiology, Nephrology and Endocrinology, Nordsjællands Hospital, Denmark. In-home assessments of OSA were performed using an ApneaLink+® device. An apnoea-hypopnoea index (AHI) ≥ 15 was categorised as OSA. An AHI < 5 was categorised as non-OSA. The inclusion criteria were as follows: patients who are ≥18 years of age, who have T2DM (WHO criteria), who have an AHI ≥ 15 or AHI < 5 measured, and who have signed informed consent. The exclusion criteria were as follows: patients with contraindications to CPAP treatment, those having treatment with CPAP within the last six months prior to inclusion, those working in a transport-related industry, those having arterial fibrillation, those with major cardiovascular event within the last three months, those with heart failure (NYHA class III or IV), those with moderate-to-severe chronic obstructive lung disease, those with other sleep breathing disorders, those who had alcohol/drug abuse, those who are pregnant or nursing women, and those who are fertile women not using contraception.

Informed consent was obtained from all patients, and the local ethical committee approved the protocol (H-D-2008-124) in accordance with the 2000 Declaration of Helsinki as well as the Declaration of Istanbul 2008.

### 2.2. Inspiratory Muscle Strength

The IMS was determined using the POWERbreathe KH2 device, and the maximal inspiratory pressure (PI_MAX_) was reported in cmH_2_O. The participants were instructed to sit in a chair and close the nose using the thumb and index fingers. Patients were instructed in a warm-up exercise in which they completed approximately seven breaths against increasing resistance. The warm-up programme was then followed by a series of at least three measurements. If the last measurement was the highest, another measurement was performed until a lower value was measured to make sure that a maximum value was reached.

### 2.3. PI_MAX_ Reference Values

The PI_MAX_ reference values were calculated using the algorithm by Evans and Whitelaw [14]. The algorithm is based on PI_MAX_ data from previous studies, which, to the knowledge of the authors from this study, included data on more than 5000 subjects with and without diseases from general populations. Using reference values that were calculated is an alternative to the inclusion of an age- and gender-matched control group which was not included in this study. The strength of the present calculated reference values is the high number of individuals from general populations, where the algorithm is based on. The following equations were used: 120 males − (0.41 × age) and 108 females − (0.61 × age). The algorithm is a linear regression reference equation as a function of age for adults aged up to 70 years [14]. A reference value was created for every patient using the patient's gender and age to match the patient.

The following equations were used to determine the lower limit of normal: 62 males − (0.15 × age) and 62 females − (0.50 × age), also as reported by Evans and Whitelaw [14].

### 2.4. Blood Pressure, Pulse, and Glycaemic Control

Blood pressure and pulse were measured twice with a 10-minute rest allowed between each measure. The measured blood pressure with the lowest diastolic value and the lowest pulse were selected. Blood pressure was reported as mmHg and pulse as beats per minute. Glycaemic control was reported as haemoglobin A1c (HbA1c).

### 2.5. Physical Activity

An accelerometer (Actical, Philips Respironics, Bend, Oregon, USA) was used to measure PA. The device is an omnidirectional, waterproof, piezoelectric accelerometer. The size is 29 × 37 × 11 mm^3^ with a weight of 16 grams. The accelerometer registers vibrations during acceleration, which produce a proportional variable electrical voltage. We reported activity counts (AC) per day as reported previously [15]. The accelerometer was placed on the patient's ankle, and the patient was instructed to wear the accelerometer for one week, except whilst showering. Data from the accelerometer were downloaded, and mean activity counts per day were calculated based on at least five days of data registration.

### 2.6. Statistical Analyses

Statistical analyses were carried out using IBM® SPSS® Statistics 19. Data distributions were tested using Q-Q plots and histograms. The parametric unpaired Student's *t*-test was used to test for differences in the two groups' continuous variables with normal distributions.

The nonparametric unpaired Mann–Whitney *U* test was used to test for differences in continuous variables without normally distributed data. Differences in categorical variables were tested using a Chi-square or Fisher's exact test. In order to adjust for differences between the groups, a multinomial linear regression analysis was performed. In the regression analysis, PI_MAX_ was included as a dependent variable and age, gender, body mass index (BMI), HbA1c, and smoking were included as independent variables, and the results are presented as *β* and 95% confidence interval (CI) The participants' characteristics are presented as the mean ± standard deviation (SD) or as median (range). All correlations were analysed using the Spearman rank correlation test for nonparametric analyses. All tests were two-tailed and significance was taken as being *p* ≤ 0.05.

## 3. Results

### 3.1. Characteristics of the Participants

Sixty-one participants were included in this study. The participants' characteristics are presented in Table 1. The percentage of women was 18% in the OSA group and 33% in the non-OSA group (*p* = 0.16). The OSA group was on average 6.6 years older than the non-OSA group (*p* = 0.001). The BMI in the OSA group was 5.3 kg/m^2^ higher than that in the non-OSA group (*p* < 0.001), and HbA1c was 7 mmol/mol higher than that in the non-OSA group (*p* = 0.007). The OSA group had higher AHI values than the non-OSA group due to the nature of the protocol. There was no difference in blood pressure between the two groups. The percentages of current smokers were 6% in the OSA group and 25% in the non-OSA group (*p* = 0.038).

### 3.2. Maximal Inspiratory Pressure

There was no difference between the OSA group's PI_MAX_ on 77 (35–124) cmH_2_O and the non-OSA group's PI_MAX_ on 84 (33–122) cmH_2_O (*p* = 0.97).

**Figure 1:**
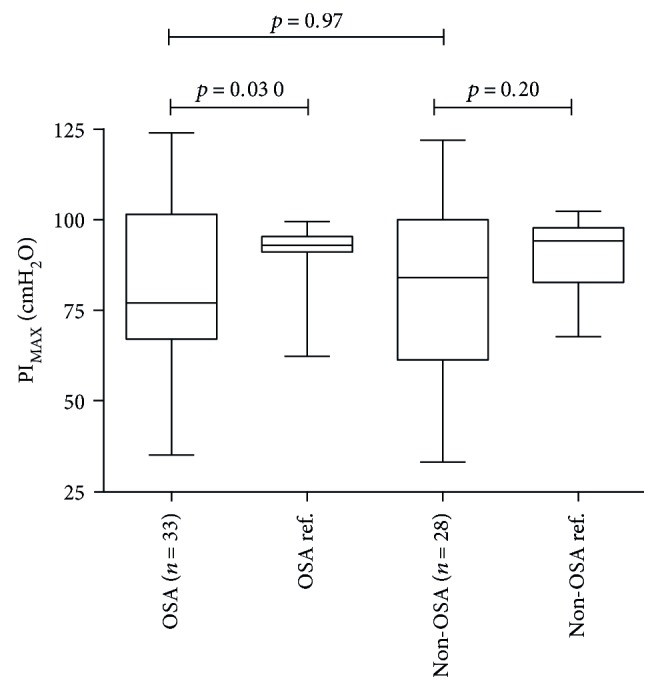
Maximum inspiratory muscle pressure. Median (range) from the two groups with their, respectively, reference values. PI_MAX_: maximal inspiratory muscle pressure; OSA: obstructive sleep apnoea.

**Figure 2:**
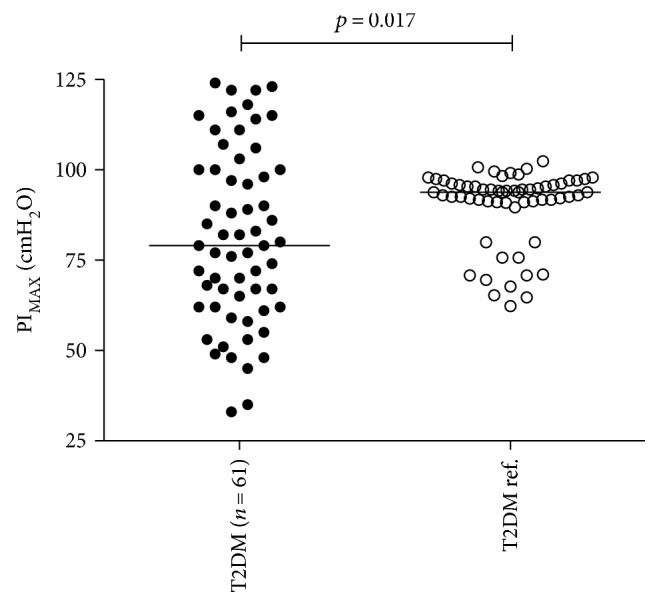
Maximum inspiratory muscle pressure. Median data from the pooled T2DM group (79 (33–124) cmH_2_O) and the reference group (93.8 (62.3–102.4) cmH_2_O). The lines in the middle represent the groups' median values. PI_MAX_: maximal inspiratory muscle pressure; T2DM: type 2 diabetes mellitus.

Whilst the PI_MAX_ values in the OSA group were reduced compared with the PI_MAX_ reference values (77 (35–124) cmH_2_O versus 92.9 (62.3–100.0) cmH_2_O, *p* = 0.030), there was no difference between the non-OSA group and the respective PI_MAX_ reference values (84 (33–122) cmH_2_O versus 94.2 (67.7–102.4) cmH_2_O, *p* = 0.20) (see Figure 1). In all patients with T2DM, the median PI_MAX_ value of 79 (33–124) cmH_2_O was different from the PI_MAX_ reference value of 93.8 (62.3–102.4) cmH_2_O (*p* = 0.017) (see Figure 2).

A negative correlation between PI_MAX_ and age was found in the OSA group (*r* = −0.483, *p* = 0.004), whereas no significant correlation was found between PI_MAX_ and age in the non-OSA group (*p* = 0.82). The PI_MAX_ was not associated with AHI in any of the groups (OSA *p* = 0.73, non-OSA *p* = 0.07).

Six out of 61 patients (10%) had a PI_MAX_ value below the lower limit of normal. The number of patients with a PI_MAX_ value below the lower limit of normal was four out of 33 patients (12%) (OSA group) versus two out of 28 (7%) (non-OSA group) (*p* = 0.52).

### 3.3. Physical Activity

Due to missing data in nine participants, PA was analysed in 52 participants. There was no difference in PA level between the two groups. The correlation between PA and PI_MAX_ in the OSA group was not significant (*r* = 0.156, *p* = 0.40).

### 3.4. Body Mass Index

There was no significant association between BMI and PI_MAX_ in the OSA group (*r* = 0.252, *p* = 0.16) or in the non-OSA group (*r* = 0.290, *p* = 0.13).

In the multinomial linear regression analysis adjusted for age, gender, BMI, HbA1c, and smoking, there was no significant association between OSA and PI_MAX_ (*β* −9.4, 95% CI −26.6; 7.9, *p* = 0.28).

## 4. Discussion

The main finding of the present study was that there was no difference between PI_MAX_ in patients with T2DM with or without OSA. However, when the two groups' PI_MAX_ values were compared with the reference values, the OSA group had reduced PI_MAX_, whereas PI_MAX_ was not reduced in the non-OSA group.

Studies have shown that an increased age has a strong negative impact on PI_MAX_ [14, 16–18]. Despite a higher age, impaired glycaemic control, and elevated BMI in the OSA group, the PI_MAX_ was not reduced compared with that in the non-OSA group as anticipated. On the other hand, the non-OSA group included relatively more women and current smokers, and whilst women in general have lower PI_MAX_ compared to men [14], smoking could also be anticipated to be associated with reduced PI_MAX_. Thus, several factors may have had influence on the result of the comparison between the two groups' PI_MAX_. After confounder control in a multinomial linear regression analysis, there was no association between OSA and PI_MAX_. It is however important to note that the result of the regression analysis was achieved on the relatively low number of subjects in the study.

In another analysis with an adjustment for the age difference in the two groups, reference values were calculated to compare the patients with the control population [14]. The OSA group's PI_MAX_ values were reduced compared with age- and gender-matched reference values, whereas the non-OSA group's PI_MAX_ values were not reduced. These results indicate that OSA is associated with reduced PI_MAX_.

The hypothesis was that PI_MAX_ was impaired in the OSA group as a result of a potential chronic overload of *m. diaphragma* working against an obstructed upper airway which could lead to a negatively altered structure in the inspiratory muscle. Furthermore, the oxidative stress that often occurs in patients with OSA could also be expected to play a role in a potential negative impact on the IMS [19]. The literature within the present research area is inconsistent, and the results vary from no difference in PI_MAX_ to a difference between groups with or without OSA. Shepherd et al. [20] investigated the relation between inspiratory pump muscle force and OSA and found no difference in PI_MAX_ in patients with and without OSA [20]. However, the study by Shepherd et al. differs from the present study in terms of not having patients with T2DM included. Mezzanotte et al. [21] found reduced PI_MAX_ in patients with OSA compared to age- and BMI-matched controls, even though the aim of the study was not to compare PI_MAX_ between the two groups. Furthermore, one should notice that the study by Mezzanotte et al. included a relatively low number (*n* = 11) of participants with OSA and 14 participants without OSA. A study by Chien et al. [12] found that people with OSA had reduced PI_MAX_ compared to people without OSA. Fifteen participants with an AHI ≥ 30 were included. The control group was matched for age, weight, and height. Whether gender was equally distributed in the two groups is unknown, and it is therefore difficult to conclude that PI_MAX_ was reduced as a result of OSA. The inconsistent results between the present and previous studies may be due to differences in patient cohorts.

The data from this study may suggest that T2DM per se was associated with reduced PI_MAX_, as one such result was found when the patient groups' values were collapsed and compared with reference values. Type 2 diabetes may lead to reduced IMS as a result of the catabolic factors associated with T2DM. This catabolic effect on muscles is induced by more complications including neuropathy, insulin resistance, and chronic low-grade inflammation and may impact the diaphragm muscle negatively. Indeed, neuropathy may play a role in the reduced PI_MAX_, as a study [22] in patients with T2DM and autonomic neuropathy found reduced PI_MAX_ in their participants. In the present study, most of the patients also suffered from OSA, and when previous and recent data are taken into account, it is difficult to see whether T2DM plays a significant role in the development of impaired IMS. Nevertheless, diabetic complications may affect the diaphragm muscle and reduce the inspiratory strength.

This study did not find any difference in PA between the OSA group and the non-OSA group, whereas a study from 2013 by Verwimp et al. found a negative correlation between PA and AHI [23]. It is, however, important to note that the PA data in the present study had a relatively wide variation, maybe due to the method used.

Only a few studies have been made to investigate the outcome of IMT in patients with OSA. Vranish and Bailey found that daily IMT for six weeks reduced the blood pressure and the patients reported fewer nighttime arousals, compared to placebo IMT [13]. In the study by Vranish and Bailey, patients with T2DM were however excluded, and it is unknown whether IMT could decrease AHI events in patients with T2DM. Our study is calling for new research to investigate the effect of IMT on patients with T2DM and OSA measured on AHI events and sleep quality.

The most important limitation of this study was the lack of a matched healthy control group. The use of reference values based on an algorithm does not replace a control group. However, the algorithm was developed on a relatively great number of subjects, a strength that may reduce biological variations in control groups. The study was also limited by a raw measure of PA and the placement of the accelerometer on the ankle. The placement on the ankle instead of the hip or thigh, which most often is used, was chosen as in Denmark, the common activity cycling may be underestimated as physical activity when accelerometers are worn on the hip or thigh [24]. Furthermore, a relatively low number of participants warrant a more comprehensive study to confirm the present findings. The results may, however, suggest that the effects of IMT should be tested on OSA in patients with T2DM, as these patients had impaired IMS values compared to reference values whereas the non-OSA patients with T2DM did not.

In conclusion, there was no difference in the IMS between patients with T2DM with or without OSA. However, patients with T2DM and OSA had reduced IMS values compared with age- and gender-matched reference values.

## Figures and Tables

**Table 1:** Characteristics of 61 patients with T2DM with or without OSA.

Variables	OSA (*n* = 33)	Non-OSA (*n* = 28)	*p*
Gender (m/f)	28/5	21/7	0.16
Age (years)	64.1 ± 6.3	57.5 ± 7.8	0.001
Weight (kg)	105.5 ± 16.2	89.6 ± 10.2	<0.001
Height (cm)	175 ± 9	173 ± 10	0.48
BMI (kg/m^2^)	34.8 ± 4.3	29.5 ± 3.3	<0.001
HbA1c (mmol/mol)	63 (43–85)	56 (43–78)	0.007
Diastolic BP (mmHg)	78 (64–92)	82 (48–95)	0.55
Systolic BP (mmHg)	142 (113–178)	139 (100–171)	0.15
Pulse (BPM)	76 (59–101)	69 (54–117)	0.07
Current smoker	2/33	7/28	0.038
AHI (events × h^−1^)	32 (15–66)	3 (0–4)	—
Physical activity (AC)	235,858 (134690–497,931)	287,060 (129127–1,148,759)	0.25

Data are presented as the mean ± SD or as counts for the normally distributed variables. Nonnormally distributed data are presented as median (range). BP: blood pressure; AC: mean activity counts per 24 hours; BMI: body mass index; AHI: apnoea-hypopnoea index.
